# Differences in bone mineral density of trajectory between lumbar cortical and traditional pedicle screws

**DOI:** 10.1186/s13018-019-1169-y

**Published:** 2019-05-09

**Authors:** Renjie Zhang, Hai Gao, Huimin Li, Tao Xing, Chongyu Jia, Jianxiang Zhang, Fulong Dong, Cailiang Shen

**Affiliations:** 1 Department of Orthopedics, The First Affiliated Hospital of Anhui Medical University, 210 Jixi Road, Hefei, 230022 Anhui China; 2 Department of Orthopedics, The First Affiliated Hospital of USTC (Anhui Provincial Hospital), Hefei, 230022 Anhui China; 3 Department of Orthopedics and Spine Surgery, The First Affiliated Hospital of Anhui Medical University, Hefei, 230022 Anhui China

**Keywords:** Cortical bone trajectory, Radiological study, CT number, Hounsfield unit

## Abstract

**Background:**

Cortical bone trajectory (CBT) has been well-known in spine surgery for obtaining improved fixation while minimizing soft tissue dissection. This study was designed to compare the bone mineral density (BMD) between the CBT and traditional trajectory (TT) by using Hounsfield unit (HU) values and identify the ideal decades of patients and the suitable lumbar segments using this CBT technology from a radiological standpoint.

**Methods:**

Patients were selected randomly from an institutional database based on age (evenly distributed by a decade of life) and gender. A total of 240 healthy patients had a computed tomography (CT) scan of the chest, abdomen, and pelvis. For each patient, axial slices of every vertebra were cut in two planes: one horizontal to the pedicle representing the plane wherein pedicle screws were inserted using the TT and the other in a caudocranial plane representing the plane wherein pedicle screws were inserted using the CBT. For each trajectory, a region of interest (ROI) was selected within the area wherein the screws were inserted. A CT number (HU values) was then calculated within each ROI to represent bone density.

**Results:**

HU values measured at the ROI of CBT were significantly greater than those of the traditional pedicle screw in all age groups, and the specific value (ratio of the HU values of CBT/the HU values of TT) between CBT and TT was 1.92. A significant difference was observed between male and female. The HU values of CBT and TT of males were generally higher than those of females (males: CBT/TT 1.89 ± 0.45; Females: CBT/TT 1.95 ± 0.47). The specific value in HU values significantly increased with increasing age (*p* = 0.000) and cauda lumbar level (*p* = 0.000) in males and females.

**Conclusion:**

BMD, as measured by HU values for the ROI of the CBT screw, was significantly greater than that of the traditional pedicle screw, especially in old patients and cauda lumbar segments.

## Introduction

Lumbar pedicle screw instrumentation has been a prop clinical surgeon technique for spinal fusion in treating a wide range of spinal disorders, such as fracture, degenerative diseases, scoliosis, kyphotic deformities, and tumors. Traditional pedicle screw fixation provides numerous advantages, the most important of which is reducing rates of loss of fixation and nonunion [1]; however, screw loosening and loss of stability of construct also occur, particularly in patients with poor bone quality [2]. Many laboratory investigations have consistently shown that osteoporotic vertebrae demonstrate reduced insertion torque and pullout strength [3–6]. To solve this challenge, an alternative to traditional pedicle screw trajectory fixation has recently been offered in lumbar fusion constructs. Cortical bone trajectory (CBT) utilizes a medialized trajectory in the caudocephalad direction to engage the cortical bone associated with pars interarticularis and pedicle [7]. The CBT technique has a theoretical benefit for patients with osteoporosis because cortical bone is less affected by the osteoporotic process than cancellous bone. A biomechanical study of CBT has revealed a 30% increase in resistance to axial pullout load compared with that in the traditional trajectory (TT) in osteoporotic cadaveric lumbar spine [7]. As a common factor used to predict fixation strength, the insertional torque using the CBT technique is 71% higher than that of the traditional technique in terms of mechanical behavior in vivo [8]. Baluch et al. [9] also examined the fixation strength of CBT screws under cyclic physiological loads and demonstrated superior resistance to craniocaudal toggling compared with that of traditional pedicle screws.

Computed tomography (CT) has become increasingly important in the field of orthopedic research in estimating bone strength, and its application in diagnosing osteoporosis and assessing fracture risks is being investigated. CT numbers, expressed in Hounsfield units (HUs), can be used to calculate bone mineral density (BMD) and estimate bone strength [10–12]. HUs have also been used to approximate regional oral BMD and have been shown to correlate strongly with insertion torque and the stability of metal implants in vitro and in vivo [13–15]. The aims of this study are to evaluate such findings from a radiological standpoint by comparing the average bone CT number (HU values) within the area wherein pedicle screws are normally placed for both trajectories and determine the optimum ages and lumbar segments.

## Materials and methods

Between December 2015 and May 2017, all healthy patients who underwent consecutive physical examinations had a CT (GE, Discovery CT 750 HD) scan of the chest, abdomen, and pelvis. Reformatted images with a thickness of 0.625 mm were obtained. Scan parameters included 120 kV, 440 mA, a 512 × 512 matrix, a layer thickness of 0.6 mm, and a pitch of 0.6 mm. Two-dimensional reconstructions were obtained in the coronal and sagittal planes. Patients were excluded if they had a spine fracture, history of spine surgery, infection, tumor, deformity, and obvious hyperostosis of the facet joint. Patients less than 20 years of age or patients with a width of pedicle less than the region of interest (ROI) were also excluded. A total of 240 patients were randomly selected for inclusion based on age and gender. Forty patients (20 of each gender) in each decade of life (from age 20 to above 70) were examined. An ROI was selected using axial slices of the lumbar vertebrae of patients for each trajectory by using GE picture archiving and communication system (General Electric Medical Systems, Milwaukee, WI, USA) to calculate an average HU value. Protocols and cadavers were used in accordance with the ethical guidelines established by the local ethics committee and were approved by the institutional review board (IRB) of the author’s affiliated institutions.

**Fig. 1:**
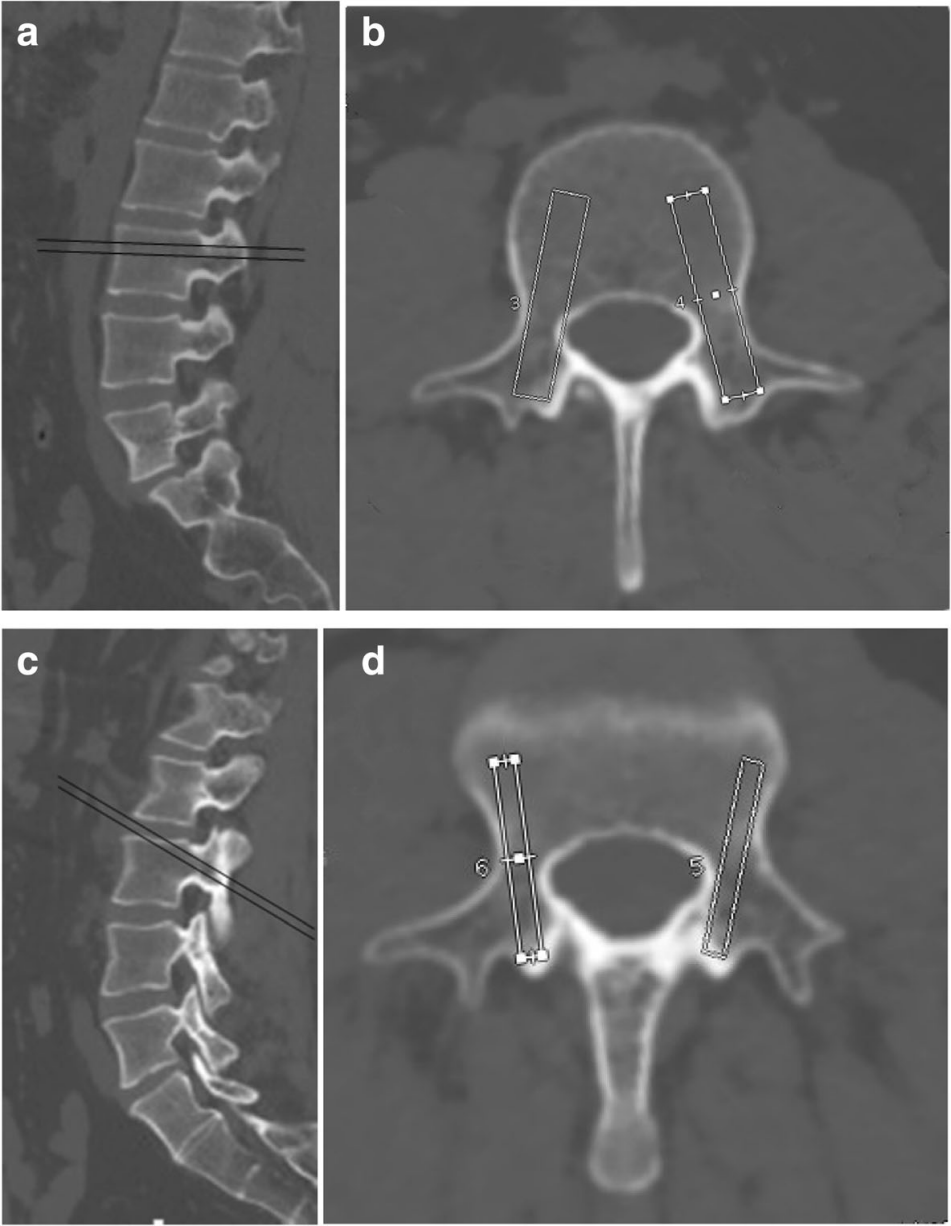
Sagittal thin-sliced CT images displaying the screw insertion angle for the TT (**a**) and CBT (**c**). ROI used for calculating the CT numbers for the TT (**b**) and CBT (**d**)

For the TT, the axial CT images were sliced in a plane horizontal to the pedicle. The ROI was fixed at 40 mm × 6.0 mm [16], which represented the pedicle screws used in the TT, and the entry point was set at the bisection of a vertical line through the facet joints and a horizontal line through the transverse process (Fig. 1a). The ROI started at this entry point and was directed toward the pedicle midline to represent the ideal area wherein to insert the screws (Fig. 1b).

For the CBT, the axial images were sliced in a caudocranial plane. The ROI was fixed at 30 mm × 4.0 mm (16), which represented the small and short pedicle screws used in the CBT. The starting point was confirmed to be the junction of the center of the superior articular process and 1 mm inferior to the inferior border of the transverse process (Fig. 1c). The ROI started at this entry point and was directed toward the pedicle midline to represent the ideal area wherein to insert the screws (Fig. 1d).

All measurements in our study were performed by two spine surgeons who were familiar with anatomy and the CBT screw insertion technique in the lumbar spine (L1–L5). Each parameter was measured thrice in each vertebra by the spine surgeons. The mean values were obtained to be the ultimate value.

### Statistical analysis

All statistics were calculated using SPSS software version 16.0 (IBM Corporation, Armonk, NY, USA). Descriptive statistics, including frequency and mean ± standard deviation (s.d.), were calculated. Paired *t* tests were used to compare paired variables between left and right pedicles and CBT and TT. The one-way test was used to compare the measured HU values among different decades and lumbar segments. Correlations between HU values and age and segment were calculated by using the Pearson correlation coefficient. Statistical significance was defined as *p* < 0.05.

## Results

The detailed information about patients' demographics is shown in Table 1. No significant difference was determined between the HU values at the ROI of the CBT and TT of left and right pedicles (CBT: *p* = 0.053, *t* = 1.935; TT: *p* = 0.474, *t* = 0.716). The mean HU values were 365.19 ± 105.05 by CBT and 199.02 ± 67.89 by TT (Table 2). The specific value (ratio of the HU values of CBT/the HU values of TT) between CBT and TT was 1.92 ± 0.46. A significant difference was found between CBT and TT (*p* = 0.00, *t* = 72.25). The correlation coefficient between CBT and TT was 0.715, which indicated a strong positive correlation. A significant difference was also presented in the HU values of CBT and TT and the specific value between CBT and TT between males and females (CBT: *p* = 0.00, *t* = 8.996; TT: *p* = 0.000, *t* = 9.672; CBT/TT: *p* = 0.024, *t* = 2.269). The specific value of CBT between males and females increased from 1.08 ± 0.27 in 20 to 29 to 1.23 ± 0.31 in above 70, the specific value of TT between males and females increased from 1.12 ± 0.32 in 20 to 29 to 1.26 ± 0.48 in above 70, and the specific value of CBT/TT between males and females increased from 0.99 ± 0.21 in 20 to 29 to 1.05 ± 0.27 in above 70.

**Table 1:** Demographics of all specimens

Age	Sex	Number	Mean age (years)	Mean height (cm)	Mean weight (kg)
20–29	Male	20	24.27 ± 3.12	164.32 ± 10.48	63.14 ± 5.17
	Female	20	25.25 ± 3.82	157.24 ± 7.14	50.45 ± 3.54
30–39	Male	20	33.82 ± 2.54	168.21 ± 8.17	66.78 ± 4.23
	Female	20	35.35 ± 3.71	160.74 ± 7.98	52.17 ± 4.51
40–49	Male	20	44.88 ± 1.88	166.87 ± 7.54	70.79 ± 6.21
	Female	20	46.46 ± 2.96	161.28 ± 6.19	61.67 ± 6.41
50–59	Male	20	54.06 ± 3.84	167.44 ± 7.46	69.18 ± 7.83
	Female	20	55.00 ± 3.16	159.97 ± 7.17	58.47 ± 5.17
60–69	Male	20	64.37 ± 2.47	166.45 ± 5.86	64.14 ± 7.41
	Female	20	64.33 ± 3.62	160.47 ± 5.24	56.71 ± 5.84
Above 70	Male	20	74.17 ± 5.47	167.32 ± 7.22	66.79 ± 5.17
	Female	20	74.00 ± 4.13	158.15 ± 6.17	56.11 ± 4.69

**Table 2:** HU values at the trajectory of CBT and TT in different decades

	Sex	Total	20–29	30–39	40–49	50–59	60–69	Above 70
CBT	M	384.75 ± 103.44	454.75 ± 83.39*^∆‡ €^	432.14 ± 74.22^†•£^	405.15 ± 85.45^Я^	379.08 ± 104.15^ØΨ^	336.86 ± 107.88	306.65 ± 76.67
	F	344.45 ± 102.84	432.68 ± 77.80*^∆‡ €^	401.93 ± 89.90^†•£^	363.64 ± 75.76^^Я^	344.20 ± 116.75^ØΨ^	289.62 ± 63.70	257.76 ± 59.71
	T	365.19 ± 105.05	444.37 ± 81.33*^∆‡ €^	417.03 ± 83.57^※†•£^	387.16 ± 83.71^^Я^	361.64 ± 111.68^ØΨ^	313.87 ± 92.03^~^	281.55 ± 72.56
TT	M	211.71 ± 64.74	278.56 ± 47.30*^∆‡ €^	254.75 ± 55.55^†•£^	225.40 ± 38.08^^Я^	197.18 ± 45.05^ØΨ^	170.64 ± 51.05^~^	148.36 ± 44.44
	F	185.56 ± 67.61	257.07 ± 47.94^#^*^∆‡ €^	214.94 ± 52.67^†•£^	209.79 ± 77.45^$^Я^	182.80 ± 54.90^ØΨ^	143.32 ± 34.67	124.98 ± 31.23
	T	199.02 ± 67.89	268.44 ± 48.6^6#^*^∆‡ €^	234.84 ± 57.54^※†•£^	218.64 ± 58.77^$^Я^	189.99 ± 50.59^ØΨ^	157.35 ± 45.839^~^	136.35 ± .9.8

Statistical significance (*p* < 0.05)

*CBT* cortical bone trajectory, *TT* traditional trajectory, *M* males, *F* females, *T* total

^#^20–29 versus 30–39 significant difference

*20–29 versus 40–49 significant difference

^∆^20–29 versus 50–59 significant difference

^**‡**^20–29 versus 60–69 significant difference

^€^20–29 versus over 70 significant difference

^※^30–39 versus 40–49 significant difference

^**†**^30–39 versus 50–59 significant difference

^•^30–39 versus 60–69 significant difference

_£_30–39 versus over 70 significant difference

^$^40–49 versus 50–59 significant difference

^^^40–49 versus 60–69 significant difference

^Я^40–49 versus over 70 significant difference

^Ø^50–59 versus 60–69 significant difference

^Ψ^50–59 versus over 70 significant difference

^~^60–69 versus over 70 significant difference

**Fig. 2:**
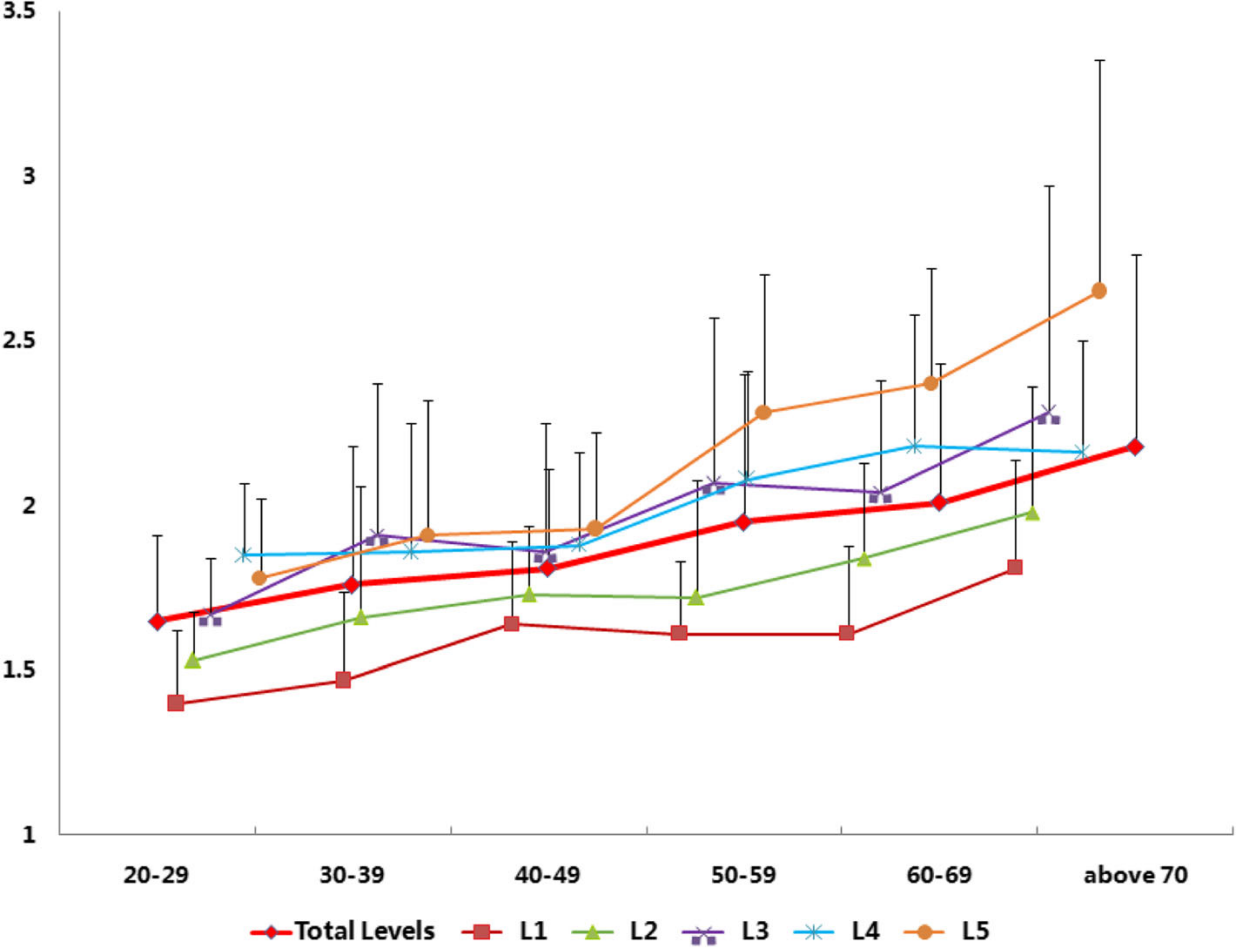
Trend of the specific value between CBT and TT (+ s.d.) of different lumbar levels in males. The average specific value between CBT and TT (+ s.d.) increased with increasing age and cauda lumbar level

For males, the HU values of CBT and TT were 384.75 ± 103.44 and 211.71 ± 64.74, and the specific value between CBT and TT was 1.89 ± 0.45. In different decades, the specific value between CBT and TT increased from 1.65 ± 0.26 in 20 to 29 to 2.18 ± 0.58 in above 70, and the correlation coefficient of the specific value between CBT and TT and decades was 0.99. The correlation coefficient of the specific value between CBT and TT and different segments was 0.97. The specific value between CBT and TT increased from 1.59 ± 0.29 in L1 to 2.16 ± 0.52 in L5 (Figs. 2 and 4). A significant difference in specific value between CBT and TT values among different decades and different segments was observed (decades: *p* = 0.000, *F* = 18.714; segments: *p* = 0.000, *F* = 32.400; Table 3).

**Table 3:** Specific value between CBT and TT in different segments on different decades of males

Segment/CBT/TT	Total	20–29	30–39	40–49	50–59	60–69	Above 70
Total	1.89 ± 0.45	1.65 ± 0.26	1.76 ± 0.42	1.81 ± 0.30	1.95 ± 0.45	2.01 ± 0.42	2.18 ± 0.58
L1	1.59 ± 0.29^#^*^∆‡^	1.40 ± 0.22^*∆‡^	1.47 ± 0.27^*∆‡^	1.64 ± 0.25^‡^	1.61 ± 0.22*^∆‡^	1.61 ± 0.27*^∆‡^	1.81 ± 0.33^‡^
L2	1.75 ± 0.34^€†•^	1.53 ± 0.15^**†**•^	1.66 ± 0.40	1.73 ± 0.21	1.72 ± 0.36^•^	1.84 ± 0.29^†•^	1.98 ± 0.38^•^
L3	1.97 ± 0.48^**£**^	1.67 ± 0.17	1.91 ± 0.46	1.86 ± 0.39	2.07 ± 0.50	2.04 ± 0.34^£^	2.28 ± 0.69
L4	2.01 ± 0.36	1.85 ± 0.22	1.86 ± 0.39	1.88 ± 0.28	2.08 ± 0.33	2.18 ± 0.40	2.16 ± 0.34
L5	2.16 ± 0.52	1.78 ± 0.24	1.91 ± 0.41	1.93 ± 0.29	2.28 ± 0.42	2.37 ± 0.35	2.65 ± 0.70

Statistical significance (*p* < 0.05)

^#^L1 versus L2 significant difference

*L1 versus L3 significant difference

^∆^L1 versus L4 significant difference

_**‡**_L1 versus L5 significant difference

^**€**^L2 versus L3 significant difference

^**†**^L2 versus L4 significant difference

^•^L2 versus L5 significant difference

^£^L3 versus L5 significant difference

**Fig. 3:**
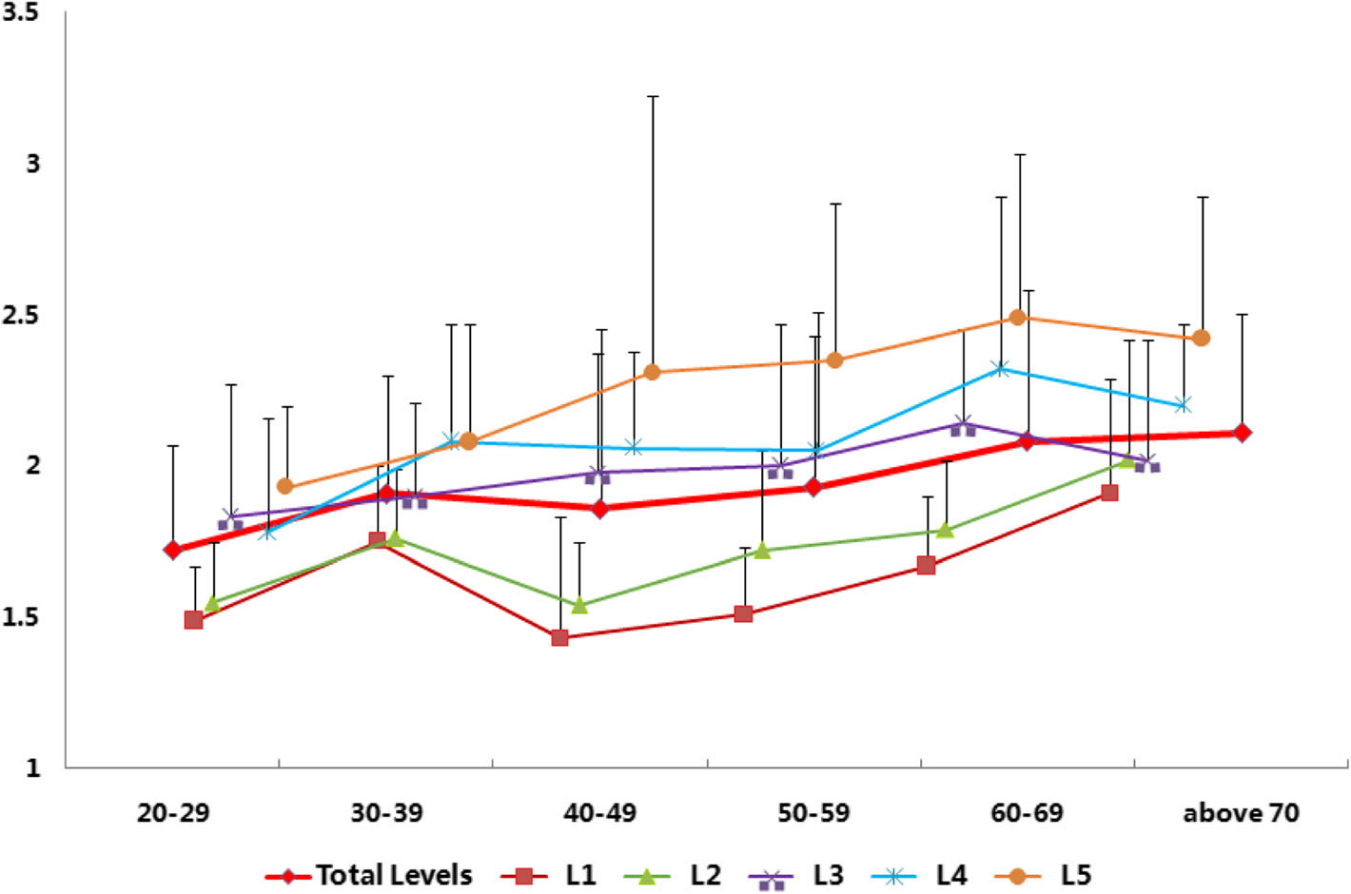
Trend of the specific value between CBT and TT (+ s.d.) of the different lumbar levels in females. Female patients experienced a notable decrease during their ages of 40 to 49, which corresponds to the age of menopause. The average specific value between CBT and TT (+ s.d.) increased with cauda lumbar level

**Fig. 4:**
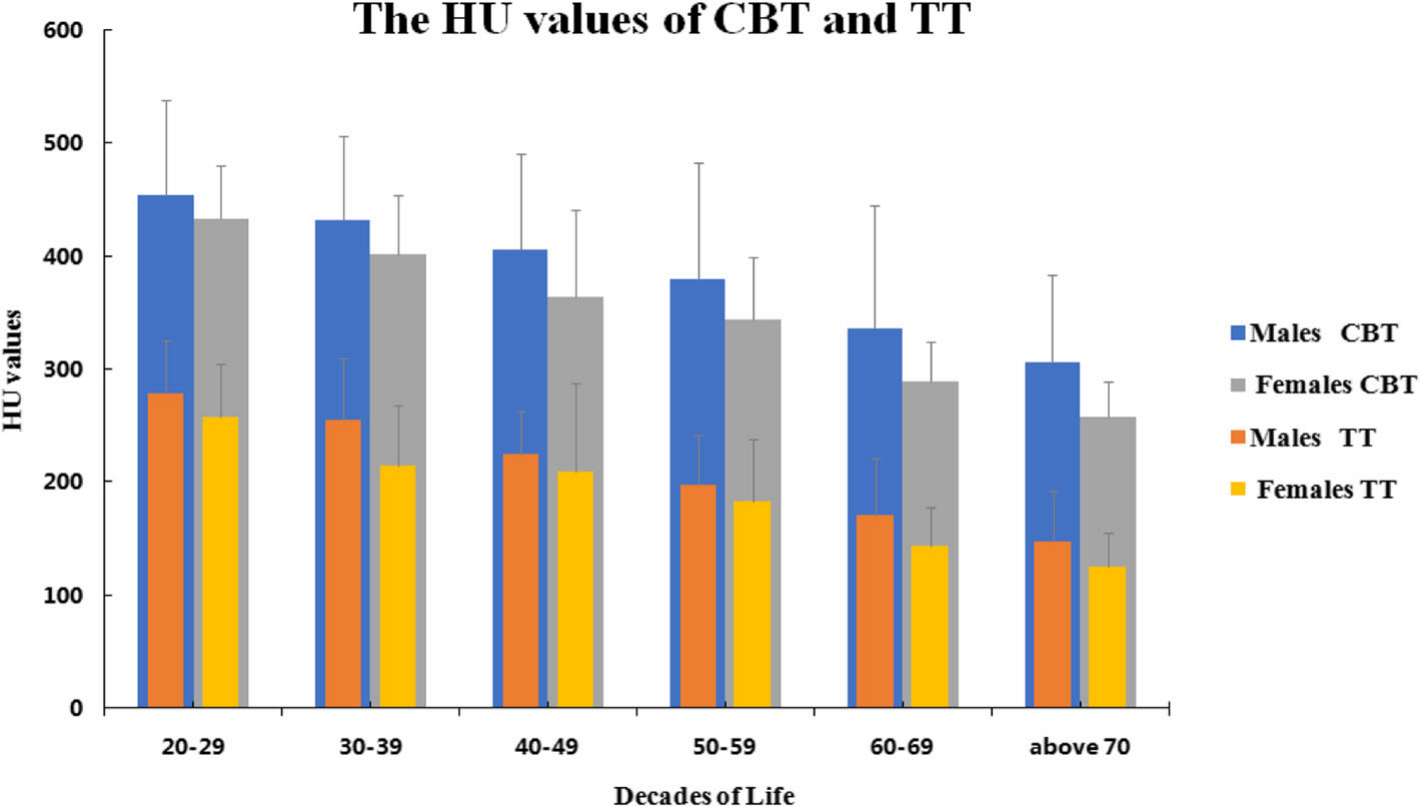
HU values of CBT and TT in different decades between males and females (+ s.d.). The HU values of CBT were larger than those of TT in males and females. The HU values of CBT and TT decreased with increasing age

For females, the HU values of CBT and TT were 344.45 ± 102.84 and 185.56 ± 67.61, and the specific value between CBT and TT was 1.95 ± 0.47. In different decades, the specific value between CBT and TT increased from 1.72 ± 0.35 in 20 to 29 to 2.11 ± 0.39 in above 70. However, females demonstrated an increasing trend with a notable inconsistency in ages 40 to 49 (*R* = 0.94). The correlation coefficient of the specific value between CBT and TT and different segments was 0.99. The specific value between CBT and TT also increased from 1.64 ± 0.33 in L1 to 2.27 ± 0.56 in L5 (Figs. 3 and 4). A significant difference in the specific value between CBT and TT values among different decades and segments was observed (decades: *p* = 0.000, *F* = 8.688; segments: *p* = 0.000, *F* = 37.313; Table 4).

**Table 4:** Specific value between CBT and TT in different segments on different decades of females

Segment/CBT/TT	Total	20–29	30–39	40–49	50–59	60–69	Above 70
Total	1.95 ± 0.47	1.72 ± 0.35	1.91 ± 0.39	1.86 ± 0.59	1.93 ± 0.50	2.08 ± 0.50	2.11 ± 0.39
L1	1.64 ± 0.33*^∆‡^	1.49 ± 0.18*^‡^	1.75 ± 0.25	1.43 ± 0.40^∆‡^	1.51 ± 0.22*^∆‡^	1.67 ± 0.23*^∆‡^	1.91 ± 0.38^‡^
L2	1.74 ± 0.32^€†•^	1.55 ± 0.20^•^	1.76 ± 0.23	1.54 ± 0.21^•^	1.72 ± 0.33^•^	1.79 ± 0.23^†•^	2.02 ± 0.40^•^
L3	1.98 ± 0.36^£^	1.83 ± 0.44	1.90 ± 0.31	1.98 ± 0.39	2.00 ± 0.47	2.14 ± 0.31	2.02 ± 0.40^£^
L4	2.09 ± 0.47^&^	1.78 ± 0.38	2.08 ± 0.39	2.06 ± 0.32	2.05 ± 0.46	2.32 ± 0.57	2.20 ± 0.27
L5	2.27 ± 0.56	1.93 ± 0.27	2.08 ± 0.39	2.31 ± 0.91	2.35 ± 0.52	2.49 ± 0.54	2.42 ± 0.47

^#^L1 versus L2 significant difference

*L1 versus L3 significant difference

^∆^L1 versus L4 significant difference

^**‡**^L1 versus L5 significant difference

^**€**^L2 versus L3 significant difference

^**†**^L2 versus L4 significant difference

^•^L2 versus L5 significant difference

^**£**^L3 versus L5 significant difference

^&^L4 versus L5 significant difference

## Discussion

Pedicle screw fixation with the traditional convergent trajectory has been a gold standard technique for spinal fixation. Although this technique has stood the test of time, it does have limitations, particularly in elderly osteoporotic and obese patients. With the traditional convergent trajectory, most of the screw thread surface rests in medullary bone, hence becoming prone to screw loosening secondary to age-related changes in bone quality and providing inconsiderably rigid fixation [7]. In obese patients with an increased depth of operating corridor, achieving a tension-free screw insertion necessitates a long incision with further lateral dissection with consequent increased blood loss, the risk of injury to the innervation of facet joints, and multifidus muscle. These limitations could potentially be overcome with a divergent trajectory (CBT) and a medial starting point. Many studies have demonstrated that the new CBT has equivalent pullout and toggle characteristics compared with the TT [7, 9, 11, 17, 18].

Several studies have correlated BMD with traditional pedicle pullout strength [8, 19]. HUs have been used to approximate regional oral BMD and have been shown to correlate strongly with insertion torque and the stability of metal implants in vitro and in vivo [13–15]. Therefore, we measured the HU values of the ROI of each trajectory to represent the BMD. While BMD has historically been assessed using dual-energy X-ray absorptiometry (DXA) and quantitative CT (qCT), the HU values from CT scans offer several benefits. HU values can be obtained throughout the entire spine, where DXA standards have not been defined. DXA results can be artificially elevated by lumbar scoliosis and degenerative changes [20, 21]. Other advantages of HU assessment over DXA assessment include the fact that HU assessment represents 3D BMD and that it can be localized to any ROI to represent the ideal trajectory; meanwhile, DXA can only measure the BMD of vertebrae, which cannot represent the trajectory. qCT examines that the bone density is the cancellous bone separate from the cortical bone in vertebrae [22–24], and the ROI of this method cannot represent the specific trajectory that combines cortical and cancellous bones. Thus, HU values can be useful in medical and surgical management.

Wray et al. illustrated that an increased bone quality was present in CBT trajectory compared with that in TT in their biomechanical study and concluded that the CBT represented a good option to obtain fixation for the lumbar spine with low-quality bone [17]. Our study kept with this conclusion from an iconography using HU values. In our study, we found that the mean HU values were 365.19 ± 105.05 on CBT and 199.02 ± 67.89 on TT, and the specific value between CBT and TT was 1.92 ± 0.46. Previous studies have reported a significant linear correlation between increasing age and decreasing BMD (evaluated by HU values) at the lumbar mid-vertebral body [12]. We also determined that the HU values of CBT and TT decreased with the increase of age. Nevertheless, the specific value between the HU values of CBT and TT increased, which may be attributed to osteoporosis more disproportionately affecting the trabecular bone than the cortical bone; thus, the fixation achieved within the vertebral body was compromised [24], and it could be explained by the fact that the CBT screws being anchored mainly in cortical bone are less influenced by the osteoporotic process than the traditional screws in cancellous bone [8].

In our study, a significant difference was observed in the HU values between male and female patients and different decades for the CBT and TT groups. Kojima et al. [16] also observed this phenomenon. However, his sub-group of decades was only over and under the age of 70 years. Female patients over the age of 70 years presented the lowest mean HU values within the ROI for CBT and TT. Mai et al. [25] concluded that the osteoporosis cohort demonstrated a significantly greater difference in HU values between CBT and TT fixation points compared with that of the non-osteoporotic cohort at all lumbar levels. The previous study also determined that the relative differences in HU values significantly increased with each decade of age at each lumbar spinal level. Furthermore, we found that the HU values of lower lumbar vertebrae were larger than those of upper lumbar vertebrae, which might be explained by the fact that cauda lumbars mostly bear the weight of activities.

Females aged 40 to 49 demonstrated an increasing trend with a notable inconsistency. The reason for this phenomenon might be the age of menopause. Similarly, Mai et al. [25] found that the relative increase in BMD between CBT and TT fixation points exhibited a notable decrease in menopause females. Schreiber et al. [26] also reported a decrease in HU-determined BMD of the lumbar vertebral bodies in females around the age of menopause.

The pedicle widths of L1 and L2 were often smaller than the ROI, which might indicate that placing CBT screws in L1 and L2 is difficult, as we also found in our previous morphometric study. Ohkawa [18] reported that the misplacing rate was approximately 4% using the CBT technique. Thus, surgeons should advance the CBT screw slowly using a C arm or CT image-guided navigation to confirm its accurate placement, and many other studies have also suggested using some tools to avoid complications [8, 27, 28].

Our study has some limitations. First, we performed our study with the Chinese. Further study should be performed to analyze whether a difference exists among human races when using CBT screws. Second, we used the ROI to represent the ideal trajectory for CBT and TT. However, actual surgical technique and trajectory may differ depending on the surgeon.

## Abbreviations

BMD: Bone mineral density
CBT: Cortical bone trajectory
CT: Computed tomography
DXA: Dual-energy X-ray absorptiometry
HUs: Hounsfield units
qCT: Quantitative CT
ROI: Region of interest
TT: Traditional trajectory
